# Interventional electrophysiology at a crossroads

**DOI:** 10.1007/s10840-021-01103-x

**Published:** 2022-01-03

**Authors:** Robert G. Hauser, William T. Katsiyiannis, Charles C. Gornick, Jay D. Sengupta, Raed H. Abdelhadi

**Affiliations:** Heart Rhythm Science Center, Minneapolis Heart Institute Foundation, 920 East 28th, Street, Minneapolis, MN 55407 USA

**Keywords:** Leadless pacemaker, Left atrial appendage closure, Devices, Quality

A major technology inflexion point has arrived with the introduction of implantable intracardiac electrophysiology devices. Currently, these include left atrial appendage closure devices and leadless right ventricular pacemakers.^1,2^ Leadless dual chamber pacing and resynchronization intracardiac pulse generators are on the horizon, and they will be integrated with subcutaneous and substernal defibrillators. The Watchman™, Nanostim™, and Micra™ experiences have taught us that intracardiac implantation requires new skills, tools, and techniques. Special training is required, and there is a learning curve for achieving the best outcomes. We also know that a small portion of implants are complicated by significant cardiac perforations that require emergency treatment.

The implantation of leadless multichamber pacing systems and defibrillators will be more challenging and require more precision than single chamber ventricular devices. Advanced multi-modal imaging will be required to facilitate pre-procedure planning and to provide precise intra-procedure navigation so that IPGs do not injure cardiovascular structures or interfere with valve function or other intracardiac devices. Some patients will undergo concomitant mapping and ablation, or have their left atrial appendages closed. The procedures and devices will require post-implant follow-up and long-term surveillance.

These new technologies have placed interventional electrophysiology at a crossroads. The question is what direction will it take? One path is to passively observe novel devices evolve on their own, as they have during the past decade, directed primarily by industry. The analysis and communication of pre-market clinical trial and post-approval registry results will be controlled solely by manufacturers whose only obligation is to report adverse clinical events to the US Food and Drug Administration (FDA). The alternative and more demanding track is for the professional cardiac electrophysiology organizations to become fully engaged at every stage of device development and to actively provide guidance and oversight. This means that the international electrophysiology community will work with industry and the appropriate regulatory authorities to achieve the safest and most satisfactory outcomes for patients.

The watch-and-wait pathway in the USA and many other countries will allow almost any licensed physician with hospital or clinic privileges to implant approved intracardiac devices upon successful completion of training programs provided and administered by manufacturers. The FDA does not have the authority to limit or dictate who performs device implants beyond that specified in the product’s labeling. Moreover, current regulations allow almost any hospital to implant intracardiac devices even though it may not have the skilled medical or surgical capabilities to effectively manage complications. Such an approach is not in the best interest of patients, and especially those at highest risk for adverse events.

Interventional electrophysiologists can benefit from the experience of their valvular heart disease (VHD) colleagues who were challenged by the increasing burden of VHD and rapidly evolving transcatheter and surgical therapies. The 2019 the Expert Consensus of Care Document^3^ for the optimal care of patients with VHD recommends the formation of multi-disciplinary Heart Valve Teams, and it describes two types of heart valve centers: level 1 is a comprehensive center that performs all interventional and surgical valve procedures, while a level 2 primary center performs the most common and least complex procedures. The centers are differentiated by their offerings and expertise, including advanced imaging, and their institution’s infrastructure and facilities. Importantly, the document emphasizes performance and outcome standards for all providers and centers.

**Fig. 1:**
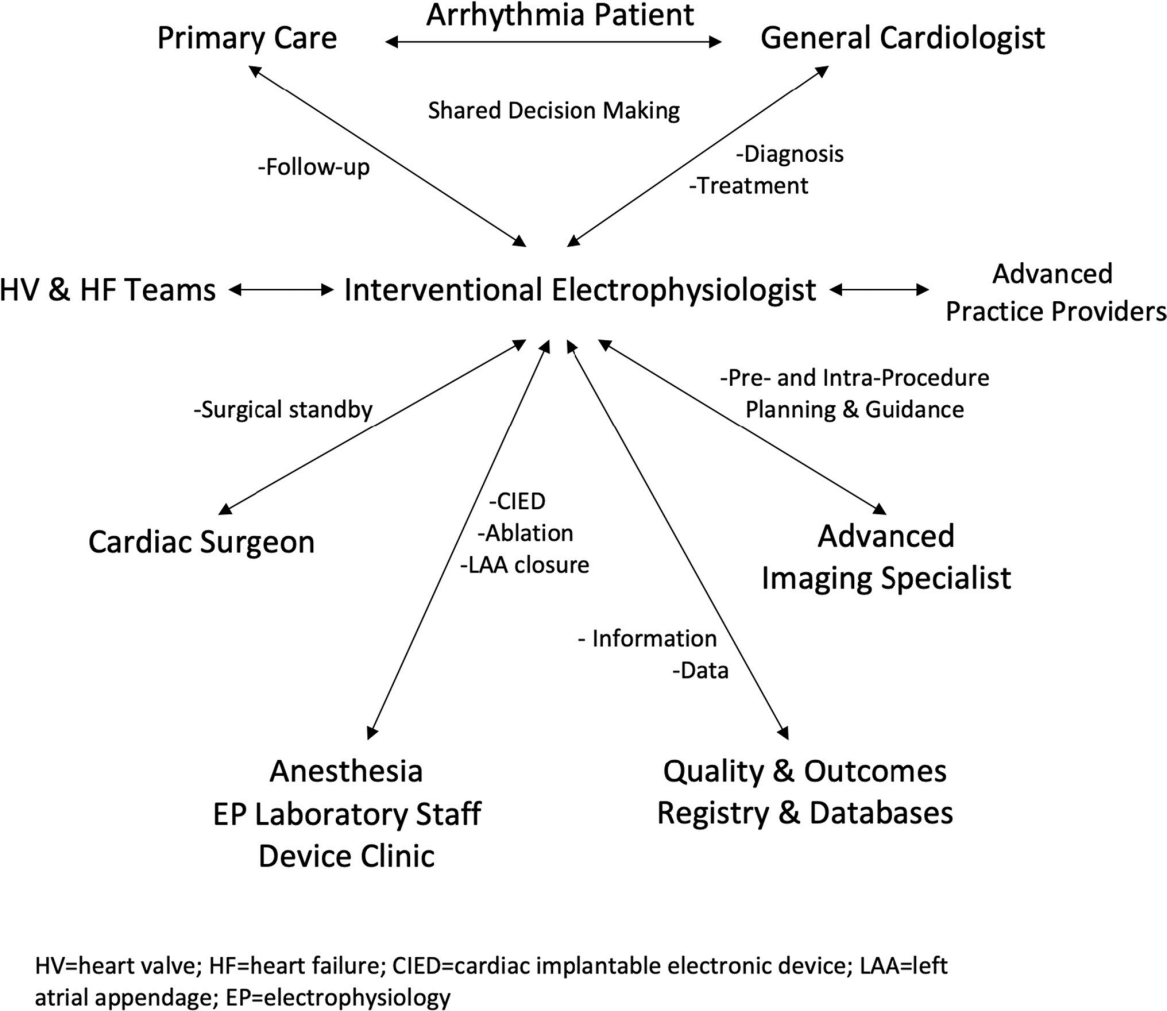
Multi-disciplinary Heart Rhythm Team

A similar approach should be taken for the treatment of heart rhythm disorders by interventional techniques. Centers should be distinguished by their methods for care delivery, procedural experience (volume and complexity), institutional competencies, and participation in clinical studies and registries. Already some electrophysiology programs have integrated multi-disciplinary teams that evaluate patients and make treatment recommendations based on clinical evidence and the patient’s wishes. The proposed multi-disciplinary Heart Rhythm Team (Fig. 1) should be a formal component of interventional electrophysiology centers.

Electrophysiologists will be confronted with difficult choices as more leadless implantable devices from multiple manufacturers enter the marketplace. Which make or model is best for a given application? What are the tradeoffs? Is one product more reliable or longer-lived than another? Are there device-to-device interactions? The answers to these questions require data, and presently electrophysiologists have no practical source other than manufacturers’ periodic product performance reports. Independent open-access registries are needed to track device performance, compare outcomes, and detect safety signals before large patient populations are needlessly exposed to the risk of preventable device-related adverse events. These online registries should be international in scope, freely accessible to participants, and led by the professional societies. We suggest the American College of Cardiology, Heart Rhythm Society, and European Society of Cardiology collaborate to create a Registry to monitor the safety and real-world outcomes of marketed leadless intracardiac devices and to publish the results regularly and when a significant safety signal is identified.

As our understanding of arrhythmias improved, so too did the technologies used to treat them. Fifty years ago, practitioners had short-lived single chamber pacemakers and mostly ineffective antiarrhythmic drugs with serious side effects. Today we employ a variety of well-studied and reliable therapies to prevent sudden cardiac death and manage almost any heart rhythm disturbance. Advances have been driven by new knowledge and technologic sophistication. However, to be safe and effective, these therapies often require multi-disciplinary expertise and a supportive infrastructure. Such attributes will be even more important in the decade ahead as new intracardiac devices and ablation techniques become widely available. Our professional organizations should collaborate to advance the practice of interventional electrophysiology so that these innovative treatments can be delivered successfully at the lowest possible risk.
